# [Corrigendum] *Macleaya cordata* extracts exert antiviral effects in newborn mice with rotavirus‑induced diarrhea via inhibiting the JAK2/STAT3 signaling pathway

**DOI:** 10.3892/etm.2024.12531

**Published:** 2024-04-04

**Authors:** Chunmao Jiang, Haifeng Yang, Xiaolan Chen, Shulei Qiu, Caihong Wu, Bin Zhang, Liqin Jin

Exp Ther Med 20:1137–1144 2020; DOI: 10.3892/etm.2020.8766

Subsequently to the publication of the above article, the authors drew to the Editor’s attention that they had made an error in compiling the data shown in Fig. 4B on p. 1141; essentially, in the first row of Fig. 4B, the pictures selected for the second and the sixth columns were the same picture, with the picture selected for the first row, sixth column (the ‘Ribavirin’ experiment) being the incorrect one. This error may have arisen inadvertently when the authors were combining the images. Moreover, the Editorial Office independently noted that the images selected for the ‘JAK2/Control’ and ‘JAK2/Rotavirus’ experiments (second row, first and second columns) appeared to contain overlapping data.

In view of the uncertainties related to the assembly of this figure, the authors were granted permission to repeat the experiments shown in Fig. 4B, and the revised version of Fig. 4, containing the new data for Fig. 4B, is shown on the next page. Note that the errors associated with the assembly of the published version of Fig. 4 did not have a major impact on either the overall results or on the conclusions reported in this study. All the authors agree with the publication of this corrigendum, and are grateful to the Editor of *Experimental and Therapeutic Medicine* for granting them the opportunity to publish this; furthermore, they apologize to the readership for any inconvenience caused.

## Figures and Tables

**Figure 4:**
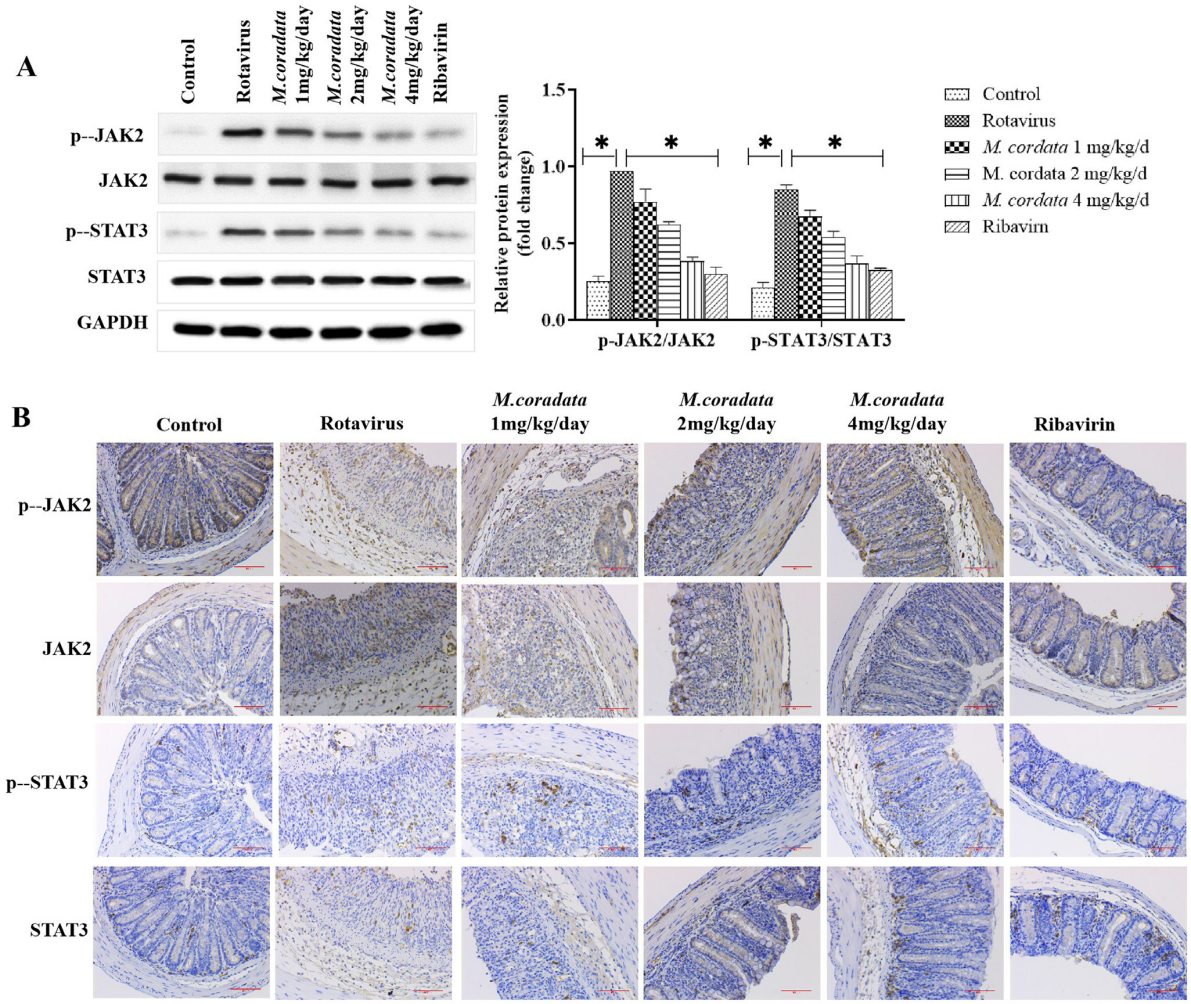
Effects of *M. cordata* on the JAK2/STAT3 pathway. (A) Protein levels of JAK2, p-JAK2, STAT3 and p-STAT3 detected by western blotting. (B) Levels of JAK2, p-JAK2, STAT3 and p-STAT3 detected by immunohistochemical analysis (magnification, x200). ^*^P<0.05. JAK2, Janus kinase 2; p, phosphorylated; t, total; *M. cordata, Macleaya cordata.*

